# A rare case of non‐lupus full house nephropathy in a transplanted kidney, case report

**DOI:** 10.1002/ccr3.8886

**Published:** 2024-05-02

**Authors:** Ahmad Samir Matarneh, Omar Salameh, Sundus Sardar, Amanda Karasinski, Theja Channapragada, Muhammad Abdulbasit, Erik Washburn, Nasrollah Ghahramani

**Affiliations:** ^1^ Department of Nephrology Penn State Milton S Hershey Medical Center Hershey Pennsylvania USA; ^2^ Department of Internal Medicine Penn State Milton S Hershey Medical Center Hershey Pennsylvania USA; ^3^ Department of Pathology Penn State Milton S Hershey Medical Center Hershey Pennsylvania USA

**Keywords:** autoimmune disorders, full house nephropathy, systemic lupus erythematosus, transplant rejection

## Abstract

**Key Clinical Message:**

Non‐lupus full house nephropathy is a rare entity that is still poorly understood. It can complicate post‐transplant kidneys and result in a de novo process. Treatment is difficult but can be possibly achieved with optimization of immune suppression.

**Abstract:**

Non‐lupus full house nephropathy is a rare entity with an unclear incidence. It describes the kidney biopsy findings of positive deposits for IgG, IgA, IgM, C3, and C1q on immunofluorescence in the absence of the classical diagnostic features of systemic lupus nephritis. This disease entity is becoming more recognized but further studies are still needed to evaluate the incidence, etiologies, and management of this condition. Transplant glomerulopathy is a major cause for renal graft loss. It can present with a wide variety of manifestations; it can cause AKI, CKD, or glomerular inflammations through an immune complex or autoimmune‐mediated damage.

## 1 INTRODUCTION

Non‐lupus full house nephropathy is defined as having the classical findings of lupus nephritis which are positive deposits for IgG, IgA, IgM, C3, and C1q on immunofluorescence but without the other extrarenal or seologic manifestations needed for SLE diagnosis i.e ACR criteria.^1^ Systemic lupus erythematosus is a multisystem autoimmune disorder. It can involve several organs with variable presentations. The diagnosis of SLE is mainly based on establishing the presence of > = 4 points of the American College of Rheumatology criteria.^2^ Lupus nephritis is the renal manifestation of SLE, it can have a wide range of manifestations which can include AKI, nephrotic, or nephritic syndrome secondary to glomerulonephritis or it can ultimately lead to ESRD. Kidney biopsy remains the gold standard test to diagnose and to guide the treatment of lupus nephritis. Classical kidney involvement in SLE is based mainly on the pattern of histological involvement, and it is divided into several classes according to the international society of nephrology.^3^ Transplant rejection remains a huge challenge to both patients and treating physicians, it can result in variable degree of kidney dysfunction and ultimately it might lead to graft loss.^4^ Several risk factors for transplant rejection have been described in literature and certainly, autoimmune diseases remain high on the list.^5^ We hereby report a patient who presented with nephrotic syndrome and was found to have de novo non‐lupus full house nephropathy in the transplanted kidney.

## 2 CASE HISTORY AND EXAMINATION

Patient is a 57‐year‐old female with a past medical history of end‐stage renal disease that was managed with a living unrelated kidney transplant in 2016, hypothyroidism, and type 1 diabetes mellitus (T1DM) managed with an insulin pump. The patient underwent a living unrelated kidney transplant in 2016 with significant post‐transplant complications with ureteral strictures. Initially, she was managed by nephrostomy followed by multiple indwelling stent exchanges. However, stents were repeatedly colonized with multiple bacterial and candida infections, requiring antibiotic, and antifungal therapy. Due to recurrent acute infections mycophenolate mofetil was held and she had a stent removal in an attempt to decrease her risk of UTI's.

She presented to an outside hospital with a 1‐week history of progressively worsening generalized headaches, nausea/vomiting, and elevated home blood pressures. Her blood pressure was noted to be 200/120. Initial laboratoties were notable for hemoglobin of 8.5 g/dL, blood urea nitrogen (BUN) of 82 mg/dL, and creatinine of 3.35 mg/dL, with baseline creatinine 2.2–2.5 mg/dL.

Physical exam on presentation was significant for +1 lower extremity edema, with the remainder of the physical exam within normal limits. Patient was afebrile and BP was noted to be 171/81 mmHg. Laboratories notable for potassium 6.1 mEq/L, chloride 116 mEq/L, bicarbonate 18 mmol/L, blood urea nitrogen (BUN) 82 mg/dL, creatinine 3.64 mg/dL, phosphorus 6.0 mg/dL, hemoglobin 8.7 g/dL, and platelets 148. VBG showed pH 7.240, PCO_2_ 41, PO_2_ 42, bicarbonate 17.6. Blood, urine, and fungal cultures were normal. A urine protein/creatinine (PCR) ratio was 12.74, consistent with nephrotic range proteinuria. (Table 1).

**TABLE 1:** Laboratory findings.

Laboratory value	Reference range	On admission	After ureteral stent	Following intravenous methylprednisone	On discharge	2 weeks after follow‐up
Hemoglobin (Hb)	12–16 g/dL (females)	8.7	7.6	9.3	8.7	9.2
Creatinine (Cr)	0.6–1.1 mg/dL (females)	3.64	3.91	3.68	2.98	2.63
Blood Urea Nitrogen (BUN)	6–24 mg/dL	82	77	105	178	134
Calcium (Ca)	8.5–10.5 mg/dL	8.4	7.7	8.0	8.5	7.9
Albumin	3.5–5.2 g/dL	2.9	2.1	2.4	3.0	3.0
Potassium (K)	3.5–5.0 mEq/L	6.1	5.3	4.9	3.9	3.9
Urine protein to creatinine ratio	<3 mg/mmol	12.74	–	–	–	5.57
Tacrolimus level	4–6 ng/mL	4.3			6.1	
C3	90–180	–	72	–	–	–
C4	10–40		16			
ANA			1:320			
Anti‐dsDNA antibody	0–200		52			
Anti‐smith antibody	<20		5.3			
ANCA			Negative			

### 2.1 Differential diagnosis and treatment

Ultrasound retroperitoneal revealed hydronephrosis in the right lower quadrant, transplant kidney with urothelial and collecting system thickening, stable from the previous ultrasound 1 month prior. Given the patient's history of prior urethral stricture, however, she had a right ureteral stent placed with the urology team. Following stent placement, however, the patient's urine output was less than 200 cc, and her creatinine rose to 3.9.

An additional renal ultrasound with doppler was done to evaluate blood flow through the transplanted kidney, revealing increased renal artery resistive indices of 0.93 and associated high resistance arterial waveform, concerning for chronic rejection. While CMV/BK and donor‐specific antibiotics were negative. Further immunological workup was done revealing positive antinuclear antibody (ANA) but negative anti‐double stranded DNA (anti‐dsDNA) and anti‐smith antibody (Sm). C3 was borderline low and C4 was normal. Additional workup for membranoproliferative glomerulonephritis, including hepatitis B and C serologies, were negative. There were still concerns about rejection, and the patient underwent a renal biopsy with interventional radiology. The preliminary read from the biopsy was concerning T cell kidney transplant rejection versus membranoproliferative glomerulonephritis versus lupus nephritis.

**FIGURE 1:**
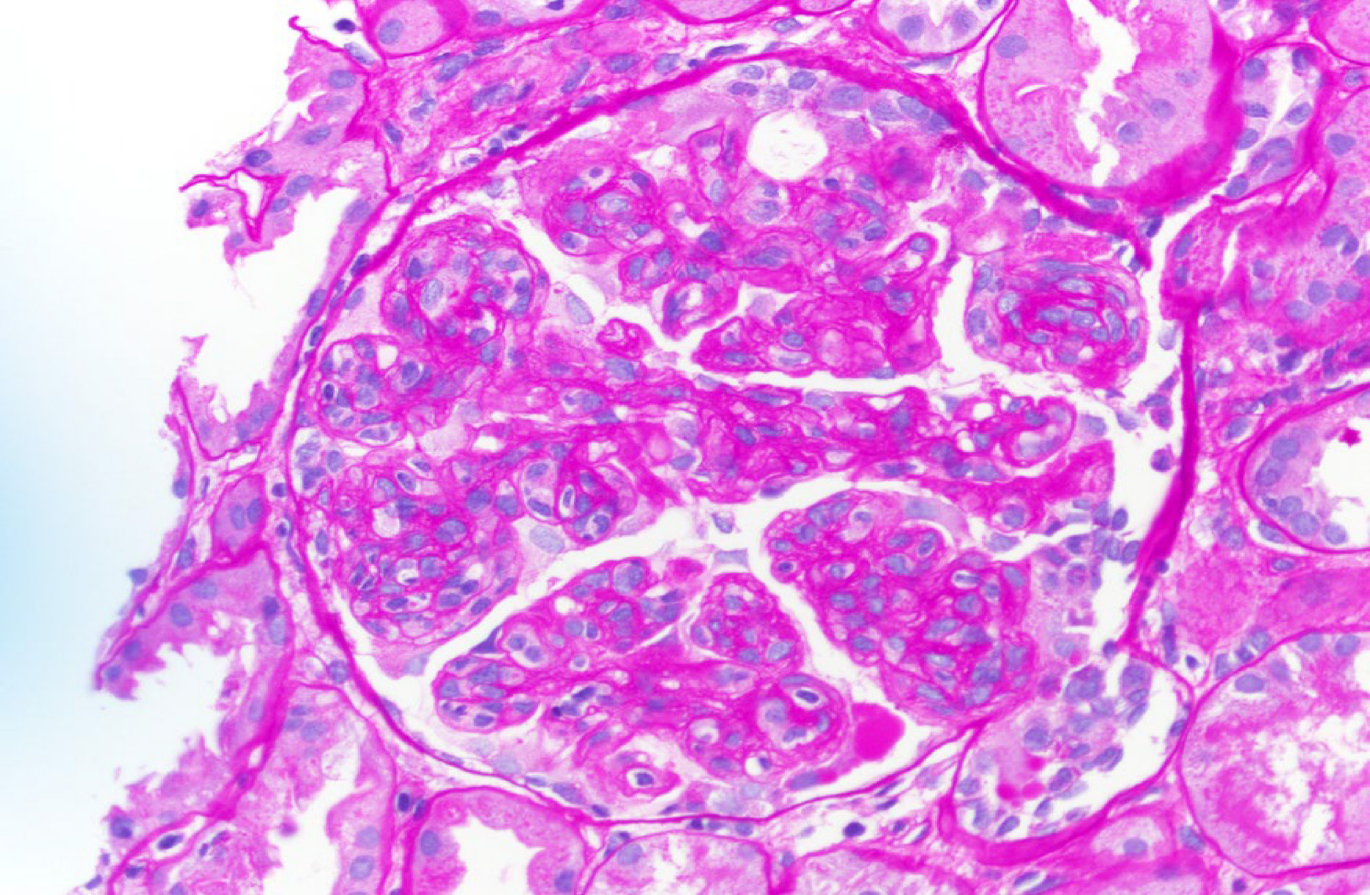
The glomeruli show diffuse and global endocapillary proliferation with lobular accentuation of the glomerular tufts. Many glomeruli are so hypercellular as to be essentially bloodless. (PAS; 400×).

**FIGURE 2:**
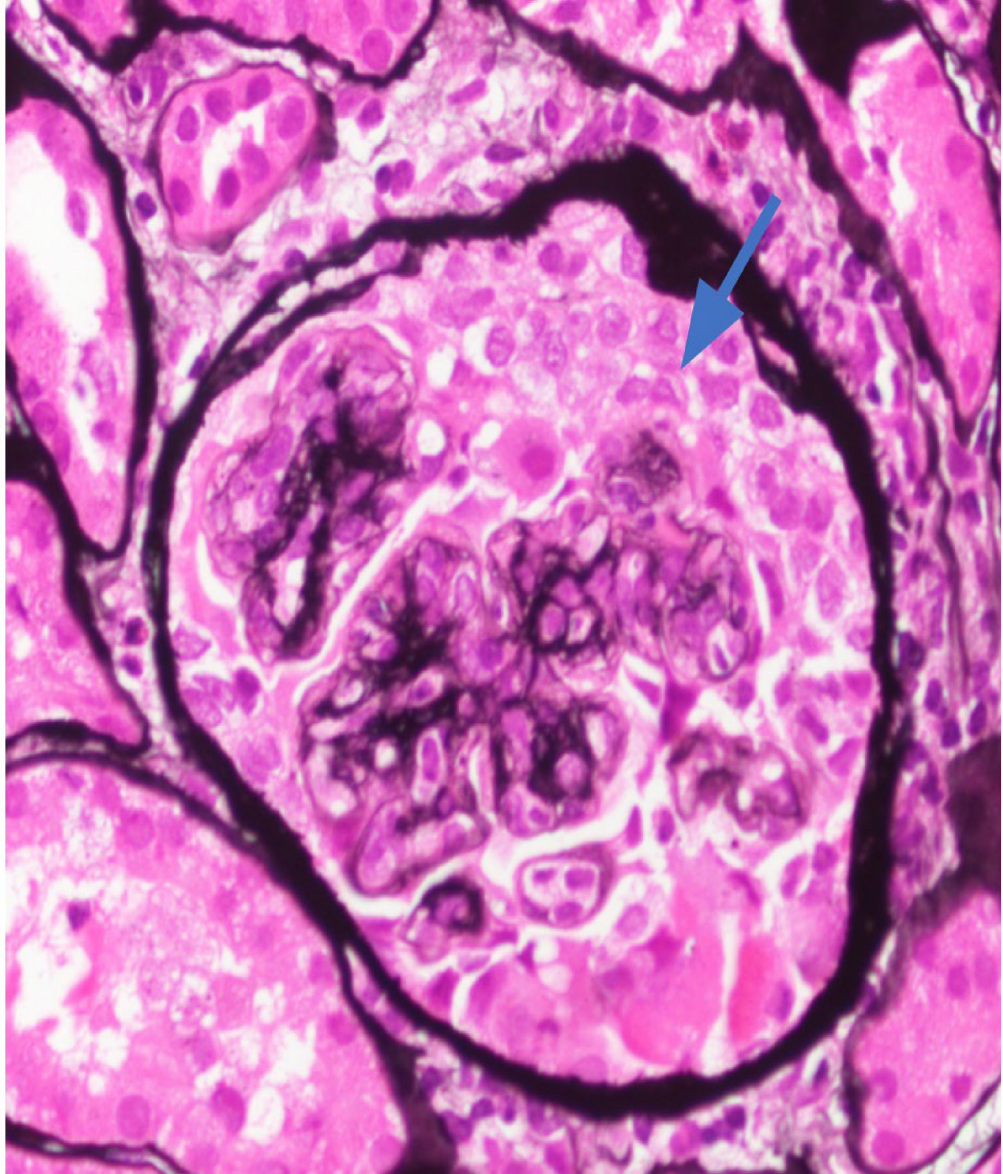
Rare cellular crescents are identified (arrow). (Jones stain; 400×).

**FIGURE 3:**
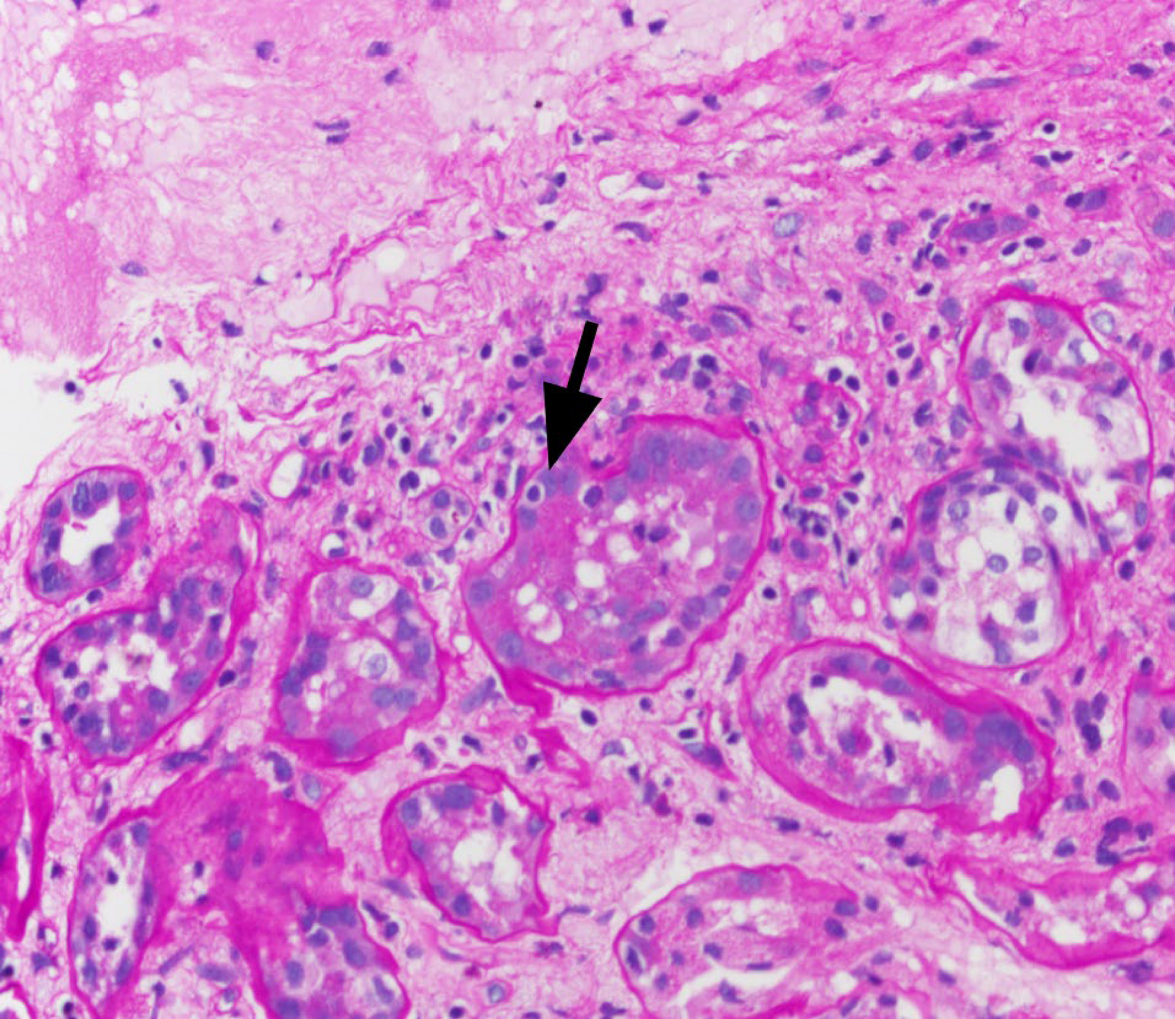
There is interstitial mononuclear cell inflammation involving nonscarred parenchyma accounting for 10%–25% nonscarred cortical parenchyma (i1). Mild tubulitis is identified in nonatrophic or mildly atrophic tubules with 1–4 mononuclear cells/tubular cross section (t1) (arrow), compatible with suspicious/borderline for acute T cell‐mediated rejection. (PAS, 400×).

**FIGURE 4:**
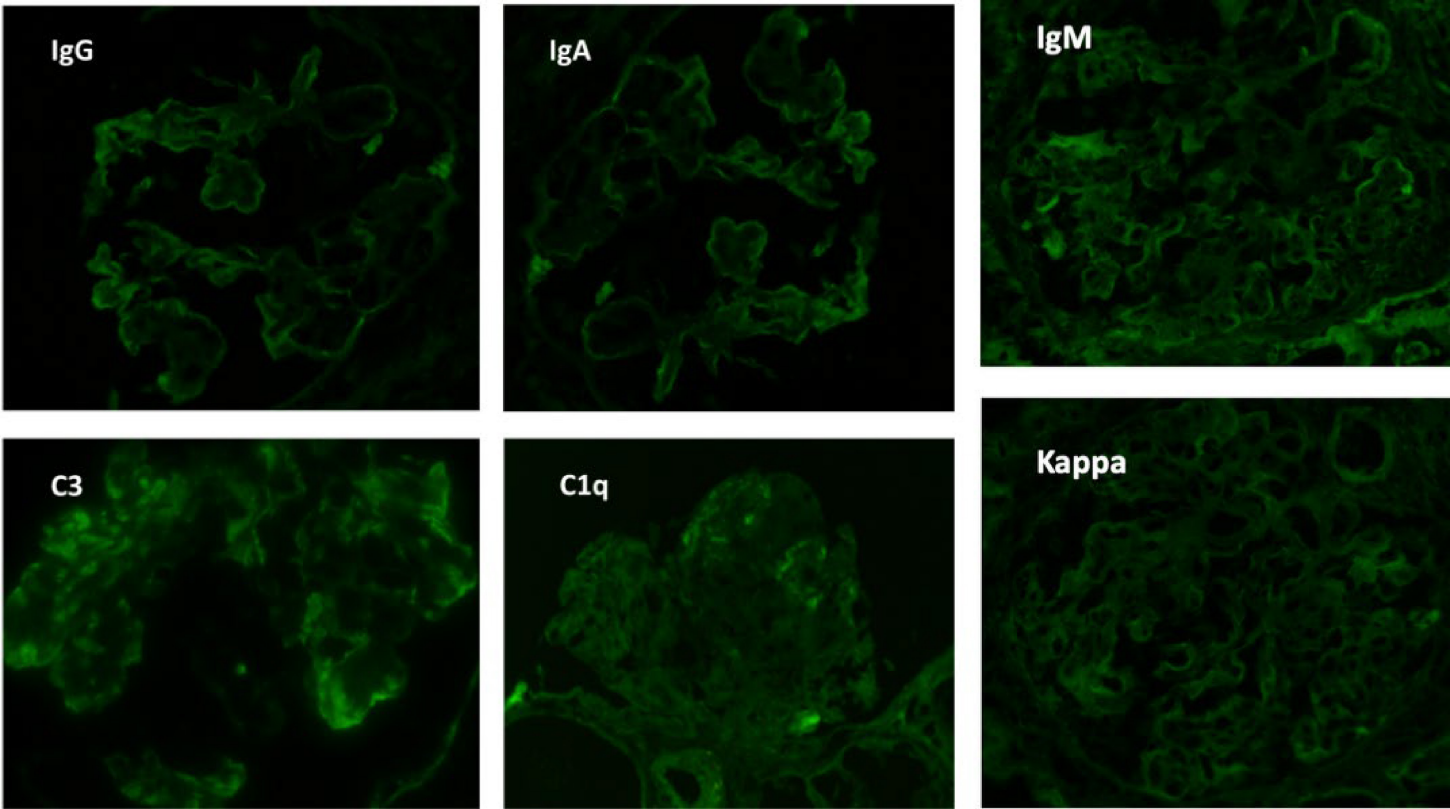
Immunofluorescence demonstrated a “full house pattern” with granular staining of the peripheral capillary wall for IgG (2+), IgA (2+), IgM (4+), C3 (4+), C1q (2+), Kappa (4+), and Lambda (4+). Albumin was negative.

**FIGURE 5:**
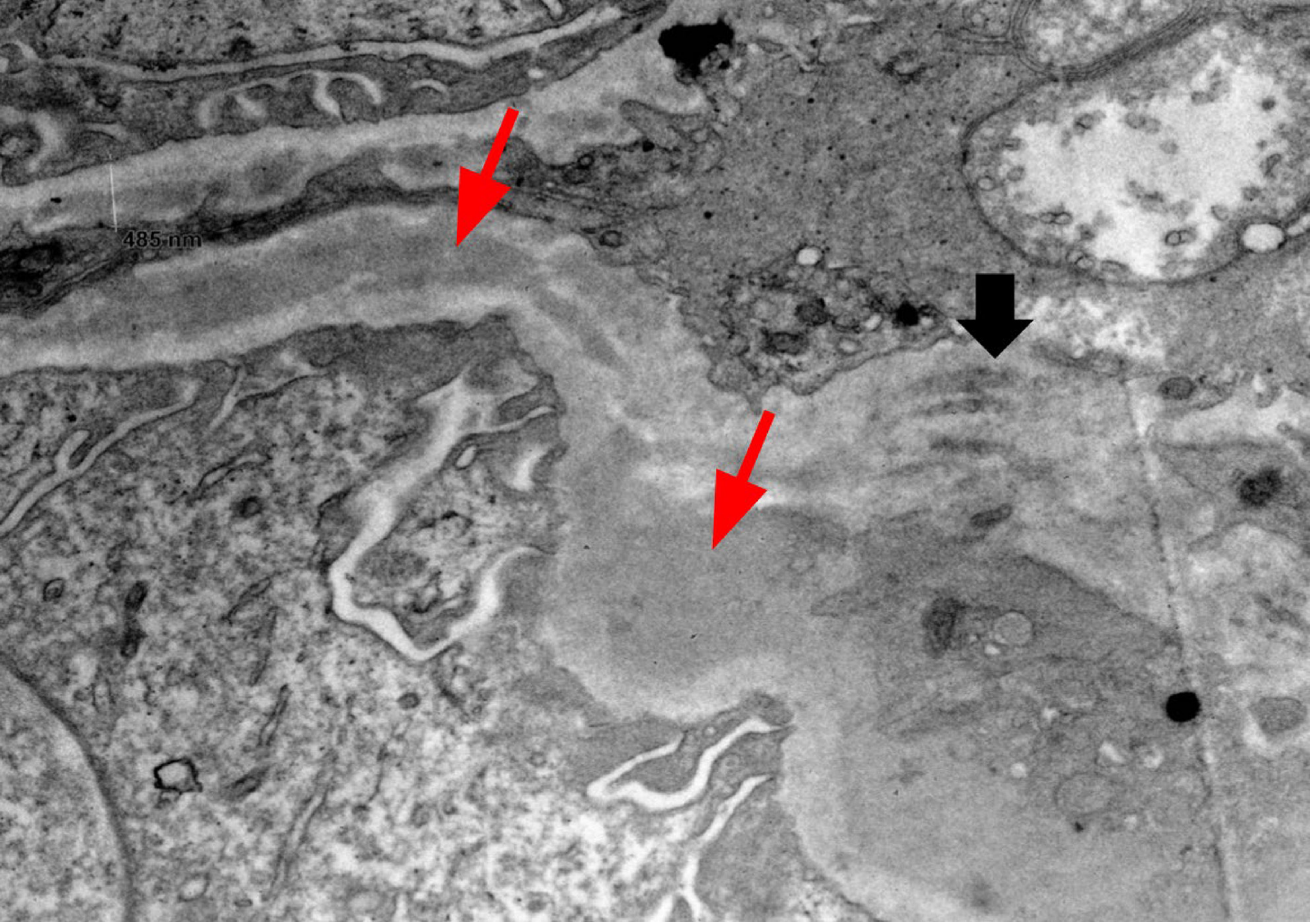
Electron microscopy identified electron dense deposits in the subendothelial space (red long arrows), with associated formation of new basement membrane material internal to the deposits (black short arrow).

The final read of the biopsy demonstrated membranoproliferative glomerulopathy, immune complex‐mediated with rare cellular crescents, and foci of fibrinoid necrosis. The full house immunofluorescence staining suggests lupus nephritis as a possible etiology. Mild tubulitis and interstitial mononuclear inflammation meet the criteria for borderline/suspicious for acute T cell‐mediated rejection. (Figures 1, 2, 3, 4, 5).

She was diagnosed as a case of chronic rejection with immune complex deposition disease. She was treated with 1 g of intravenous methylprednisolone once and transitioned to 60 mg oral prednisone once daily throughout the remainder of the course and at discharge. Her BUN continued to rise in the setting of steroid use, as high as 178 mg/dL at discharge. In addition, patient developed hypoalbuminemia with associated anasarca. Her anasarca was managed with a bumetanide drip that was transitioned to intermittent dosing of 2 mg bumetanide on discharge. The patient's urine output increased to over 1 L with diuretics, with creatinine levels down trending. On discharge, the patient's creatinine was 2.98 mg/dL.

### 2.2 Outcome and follow‐up

The patient was followed up in the transplant nephrology clinic 2 weeks after discharge. Creatinine improved to 2.63 mg/dL. Proteinuria decreased to 5.7 with a 50% reduction from hospital admission. Patient's lower extremity swelling decreased significantly. She was maintained on tacrolimus 2 mg twice daily, MMF 500 mg twice daily. Given the biopsy findings and her improvement of her creatinine and proteinuria degree it was decided to continue treatment as an underlying chronic rejection.

## 3 DISCUSSION

Glomerulonephritis remains one of the leading causes of renal graft loss following kidney transplant.^6^ The incidence is widely variable and differs between studies. Recurrent and de novo glomerulonephritis following kidney transplant are not uncommon and have been described as a cause of renal graft loss and/or dysfunction.

Recurrence of GN is typically more common than de novo processes.^7^ GN is one of the main causes of renal graft loss after kidney transplant, according to one of the studies it was noted that recurrence of GN was the third most common cause of renal graft loss.^8^ De novo disease is defined as any new disease process that occurs in the kidney graft unrelated to the primary kidney disease. De novo disease can have a variable presentation as it can manifest as acute, subacute to chronic with manifestations usually arising from glomerulonephritis, tubulointerstitial nephritis, or vascular disease. The incidence of de novo GN is unclear and has been hypothesized to be occurring in 4%–20% of transplant recipients.^9^ Most common patterns of GN are FSGS, MGN, and MPGN.^10^ Transplant glomerulopathy remains one of the major debilitating conditions that transplant patients deal with. It can present with a chronic or acute rejection picture in the form of steady rise in creatinine and GN pattern of injury.^11^ Systemic lupus nephritis is a chronic inflammatory autoimmune condition that can affect multiple organs with variable presentations. Diagnosis is usually based on the classical serologic and extra renal criteria.^12^

Lupus nephritis is one of the serious manifestations of SLE, it is defined as the presence of significant proteinuria and classical findings on kidney biopsy. Classical kidney biopsy findings of lupus nephritis under light microscopy are I. minimal mesangial, II. Proliferative mesangial, III. Focal proliferative, IV. Diffuse proliferative, V. membranous, and VI. Sclerosing. One of the relatively specific findings on direct immunofluorescence on kidney biopsy is finding deposits positive for IgG, IgA, IgM, C3, and C1q, a pattern that is usually referred to as the full house pattern.^13^ This finding is relatively specific for lupus nephritis, however, in order to establish the diagnosis of SLE, the ACR criteria must be met.^14^ Non‐lupus nephritis describes the classical IF findings of LN but without the clinical or serological evidence of lupus. It is a newly developing entity and that means further studies are still needed to better understand the incidence and its exact pathophysiology and presentations.^15^

Kidney transplant remains the gold standard treatment for ESRD. It has shown to improve both quality of life and survival in comparison to renal replacement therapies.^16^ Transplant rejection occurs as a result of the body's own immune system creating antibodies against the alloantigen from the transplanted kidney.^17^ The response can be variable in intensity and onset, it ranges from hyperacute which can happen within minutes of transplant and is usually related to preformed antibodies or ABO mismatch. Acute rejection occurs any time following transplant and is subclassified depending on the underlying factor and immune mechanism to antibody‐mediated rejection (ABMR), which usually happens as a result of donor‐specific alloantibodies damaging the kidney causing peritubulitis/capillaritis. And acute T‐cell‐mediated rejection (TCMR), which is characterized by lymphocytic infiltrate into tubules, interstitium, and arterial intima. Chronic rejection, on the other hand, usually occurs after 3 months and can be as a result of chronic T cell‐mediated or chronic antibody‐mediated.^18^

Risk factors for transplant rejection include: HLA mismatch, Positive B cell crossmatch, advanced age of the donor type of transplant, and inadequate immunosuppression.^19^ Patients with rejection present differently, any increase in creatinine more than 25% of the baseline or the presence/worsening of proteinuria should raise the suspicion for rejection. Kidney biopsy might be warranted if transplant rejection is suspected as it would guide the management and further prognosis.^20^

There has been one case in literature describing the presence of non‐lupus full house nephropathy in the setting of post kidney transplant, we hypothesize that it might be related to circulating immune complex‐mediated damage to the transplant kidney possibly in the setting of chronic active rejection. The point of interest in this condition and in our case in particular is that the patient was treated mainly for the possible underlying rejection with resuming mycophenolate and optimizing tacrolimus with institution steroids which lead to improvement in her kidney parameters and more than 50% drop in the degree of proteinuria. This further suggests that the possible underlying mechanism is related to the rejection process causing the non‐lupus pattern and nephrotic syndrome.

## 4 CONCLUSION

Non‐lupus full house nephropathy is rare and is poorly understood. It has been described in association to several conditions; however, there has been no data describing the associated post kidney transplant and rejection. Non‐lupus full house nephropathy can occur in the setting of kidney transplant and it might be related to transplant rejection. We wanted to raise awareness about this condition and possible association in transplant recipients as it carried out a worse prognosis and can lead to loss of graft function, also, to describe the methods in the diagnosis and a proposed treatment regimen.

## AUTHOR CONTRIBUTIONS

**Ahmad Samir Matarneh:** Data curation; writing – original draft; writing – review and editing. **Omar Salameh:** Writing – original draft. **Sundus Sardar:** Writing – original draft. **Theja Channapragada:** Writing – original draft. **Erik Washburn:** Writing – original draft. **Muhammad Abdulbasit:** Writing – original draft; writing – review and editing. **Amanda Karasinski:** Writing – original draft. **Nasrollah Ghahramani:** Writing – original draft; writing – review and editing.

## FUNDING INFORMATION

The funding process is solely done from the writing authors.

## CONFLICT OF INTEREST STATEMENT

The authors associated with this case report have no actual or possible conflict of interest to declare.

## CONSENT

Written informed consent was obtained from the patient to publish this report in accordance with the journal's patient consent policy.

## Data Availability

The data that support the findings of this study are available on request from the corresponding author.

## References

[1] Silva MD , Oliveira PV , Vale PH , et al. Non‐lupus full‐house nephropathy: a case series. J Bras Nefrol. 2020;11(43):586‐590. 10.1590/2175-8239-JBN-2019-0242 PMC8672396 33179718

[2] Mok CC , Lau CS . Pathogenesis of systemic lupus erythematosus. J Clin Pathol. 2003;56(7):481‐490. 12835292 10.1136/jcp.56.7.481 PMC1769989

[3] Ayoub I , Cassol C , Almaani S , Rovin B , Parikh SV . The kidney biopsy in systemic lupus erythematosus: a view of the past and a vision of the future. Adv Chronic Kidney Dis. 2019;26(5):360‐368. 31733720 10.1053/j.ackd.2019.08.015

[4] Vaillant AA , Mohseni M . Chronic Transplantation Rejection. StatPearls Publishing; 2023. 30571056

[5] Neuberger J . Incidence, timing, and risk factors for acute and chronic rejection. Liver Transpl Surg. 1999;5(4 Suppl 1):S30‐S36. 10431015 10.1053/JTLS005s00030

[6] Allen PJ , Chadban SJ , Craig JC , et al. Recurrent glomerulonephritis after kidney transplantation: risk factors and allograft outcomes. Kidney Int. 2017;92(2):461‐469. 28601198 10.1016/j.kint.2017.03.015

[7] Jafari R , Mehrazma M , Vahedi M , Ossareh S . Prevalence and prognosis of post‐transplant glomerulonephritis in kidney transplant biopsies, a single‐center report. Iran J Kidney Dis. 2023;17(2):92. 37060343

[8] Briganti EM , Russ GR , McNeil JJ , Atkins RC , Chadban SJ . Risk of renal allograft loss from recurrent glomerulonephritis. N Engl J Med. 2002;347(2):103‐109. 12110738 10.1056/NEJMoa013036

[9] Abbas F , El Kossi M , Jin JK , Sharma A , Halawa A . De novo glomerular diseases after renal transplantation: how is it different from recurrent glomerular diseases? World J Transplant. 2017;7(6):285‐300. 29312858 10.5500/wjt.v7.i6.285 PMC5743866

[10] Hariharan S , Adams MB , Brennan DC , et al. Recurrent and de novo glomerular disease after renal transplantation: a report from renal allograft disease registry (RADR). Transplantation. 1999;68(5):635‐641. 10507481 10.1097/00007890-199909150-00007

[11] Filippone EJ , McCue PA , Farber JL . Transplant glomerulopathy. Mod Pathol. 2018;31(2):235‐252. 29027535 10.1038/modpathol.2017.123

[12] Gill JM , Quisel AM , Rocca PV , Walters DT . Diagnosis of systemic lupus erythematosus. Am Fam Physician. 2003;68(11):2179‐2187. 14677663

[13] Musa R , Brent LH , Qurie A . Lupus Nephritis. StatPearls Publishing; 2023. 29762992

[14] Anders HJ , Saxena R , Zhao MH , Parodis I , Salmon JE , Mohan C . Lupus nephritis. Nat Rev Dis Primers. 2020;6(1):8. 31974366 10.1038/s41572-019-0141-9

[15] Wani AS , Zahir Z , Gupta A , Agrawal V . Clinicopathological pattern of non‐lupus full house nephropathy. Indian J Nephrol. 2020;30(5):301‐306. 33707816 10.4103/ijn.IJN_91_18 PMC7869643

[16] Braun MM , Khayat M . Kidney disease: end‐stage renal disease. FP Essent. 2021;509:26‐32. 34643362

[17] Halloran PF , Einecke G , Sikosana ML , Madill‐Thomsen K . The biology and molecular basis of organ transplant rejection. Handb Exp Pharmacol. 2022;29:1‐26. 10.1007/164_2021_557 35091823

[18] Wood KJ , Goto R . Mechanisms of rejection: current perspectives. Transplantation. 2012;93(1):1‐10. 22138818 10.1097/TP.0b013e31823cab44

[19] Oweira H , Ramouz A , Ghamarnejad O , et al. Risk factors of rejection in renal transplant recipients: a narrative review. J Clin Med. 2022;11(5):1392. 35268482 10.3390/jcm11051392 PMC8911293

[20] Williams WW , Taheri D , Tolkoff‐Rubin N , Colvin RB . Clinical role of the renal transplant biopsy. Nat Rev Nephrol. 2012;8(2):110‐121. 22231130 10.1038/nrneph.2011.213 PMC3716017

